# Comparison of coronal alignment measured on postoperative short-leg and standing long-leg radiographs following total knee arthroplasty

**DOI:** 10.1051/sicotj/2026079

**Published:** 2026-10-26

**Authors:** Rory Schanknecht, Carel Willemse, Bakatuasa Freddy Mulunda, Adriaan de Villiers, Gopika Ramkilawon, Hans Jacobs

**Affiliations:** 1 Steve Biko Academic Hospital Pretoria South Africa; 2 Dr. George Mukhari Academic Hospital Ga-Rankuwa South Africa; 3 University of Pretoria South Africa; 4 Steve Biko Academic Hospital and Life Faerie Glen Hospital Pretoria South Africa

**Keywords:** Total knee arthroplasty, Coronal alignment, Short-leg radiograph, Long-leg radiograph, *β* angle

## Abstract

*Introduction*: Coronal alignment following total knee arthroplasty (TKA) is commonly assessed using radiographs, but postoperative radiographic protocols are not standardised. This study evaluated agreement between coronal alignment measured on short-leg and standing long-leg radiographs. We hypothesised that, in knees demonstrating varus alignment on standing long-leg radiographs, short-leg radiographs would demonstrate greater apparent varus. *Methods*: This retrospective cross-sectional study included 62 sequentially selected patients following primary TKA. All included patients had both postoperative short-leg and standing long-leg radiographic examinations available for analysis. Coronal alignment was assessed using the *β* angle. Agreement was evaluated using paired differences and Bland–Altman analysis. Interobserver reliability was assessed across six observers (two primary observers and four additional colleagues) using ICC(2,6). Intraobserver reliability was assessed using ICC(3,1). *Results*: The mean paired difference (short-leg minus standing long-leg) was −1.13° (95% confidence interval [CI]: −1.40° to −0.85°), indicating a systematic shift towards a more varus measurement on short-leg radiographs. The 95% limits of agreement were −4.08° to –1.82°, and 29% of measurements differed by more than 2°. In the 47 varus knees, the mean difference was −0.79° (95% CI: −1.18° to −0.40°; *p* < 0.001). In the valgus subgroup (*n* = 14; one neutral knee excluded), the mean difference was −2.31° (95% CI: −3.22° to −1.40°; *p* < 0.001). *Discussion*: Short-leg and standing long-leg radiographs demonstrate systematic differences in postoperative coronal-alignment measurement. Although the overall mean difference was modest, the relatively wide limits of agreement indicate that larger discrepancies can occur in individual patients. Short-leg radiographs should not be considered interchangeable with standing long-leg radiographs when accurate assessment of the lower-limb mechanical axis is required.

## Introduction

Coronal alignment following total knee arthroplasty (TKA) remains an important consideration, as postoperative malalignment may adversely affect clinical outcomes and implant survivorship [1]. Traditionally, restoration of the mechanical axis to within ±3° of neutral has been considered an acceptable target following TKA [1]. Accurate postoperative assessment of coronal alignment is therefore important; however, radiographic protocols for assessing alignment following TKA are not universally standardised.

Standing long-leg radiographs are commonly used to assess postoperative mechanical alignment because they allow visualisation of the mechanical axis of the entire lower limb. Short-leg radiographs are more readily available but may not accurately represent overall coronal alignment (Figure 1). Petterwood et al. reported differences of up to 5° in 9% of patients [2]. Park et al. found discordant classification of coronal alignment as varus, neutral or valgus in approximately one-third of patients [3]. Kazemi et al. reported neutral alignment in 53.8% of postoperative short-leg radiographs compared with 28.6% of full-length radiographs [4].

**Figure 1:**
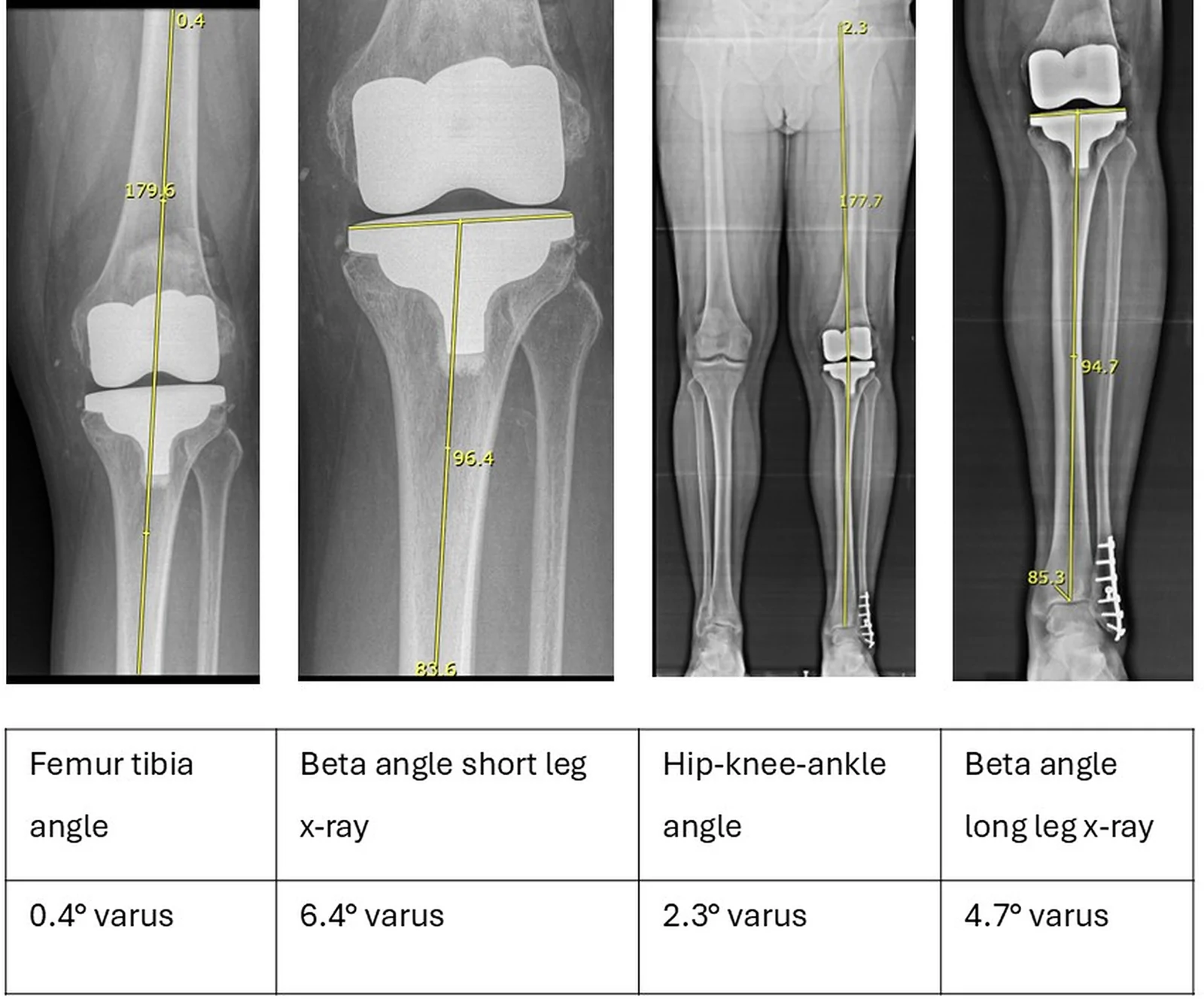
Image and table illustrating different ways of measuring alignment and how each technique has a different value when measured on the same patient.

Variation in limb positioning, rotation and radiographic technique may influence apparent prosthetic-component alignment and contribute to discrepancies between short-leg and standing long-leg measurements. The primary aim of this study was to compare coronal alignment measured on postoperative short-leg radiographs with that measured on standing long-leg radiographs following primary TKA and to quantify agreement between the techniques. We hypothesised that, in knees demonstrating varus alignment on standing long-leg radiographs, short-leg radiographs would demonstrate greater apparent varus. A secondary objective was to determine whether these findings could inform standardisation of postoperative radiographic assessment.

## Materials and methods

### Study design and patient selection

This retrospective cross-sectional study reviewed postoperative radiographs obtained from the electronic radiographic database of a private hospital in Pretoria, South Africa. Sixty-two sequentially selected patients who underwent primary TKA between 2015 and 2023 were included.

Patients of any age or sex who underwent primary TKA for osteoarthritis were included. Patients who underwent TKA for indications other than osteoarthritis or revision arthroplasty were excluded. Both kinematically and mechanically aligned knees were included. All 62 included patients had both postoperative short-leg and standing long-leg radiographic examinations available for analysis.

### Radiographic technique

All radiographs were obtained in the same radiology department using standardised imaging protocols. Short-leg radiographs were obtained with the patient supine and non-weight-bearing. The knees and ankles were positioned together, rotation was corrected at the hip and knee, and the feet were stabilised with the toes directed vertically. The beam was centred over the patella. No correction was made for fixed flexion contractures or severe deformities that prevented standard positioning. Exposure was 60 kVp and 8 mAs, with a source-to-image distance of 100 cm.

Standing long-leg radiographs were obtained as weight-bearing teleroentgenograms. Patients were positioned with the ankles together and toes facing forward, and the beam was centred over the patella. Blocks were used where necessary to compensate for leg-length discrepancy. The source-to-image distance was 180 cm; image stitching was not used. Exposure was 70 kVp and 10 mAs.

### Measurement of coronal alignment

The *β* angle was selected because it could be measured on both radiographic examinations and has been used in radiographic assessment after TKA [5–8]. On standing long-leg radiographs, the *β* angle was measured between a line from the midpoint of the tibial plafond to the midpoint of the tibial component and a line along the superior surface of the tibial component [6]. On short-leg radiographs, where the tibial plafond was not visible, it was measured between the anatomical axis of the visible tibial shaft and a line along the superior surface of the tibial component [7]. An angle of 90° represented neutral alignment, <90° varus alignment and >90° valgus alignment.

### Measurement procedure and reliability

Short-leg and standing long-leg radiographs were independently assessed by two orthopaedic surgeons. Radiographs were de-identified, sequentially numbered and measured anonymously. The measurements from the two primary observers were averaged for the comparison of radiographic techniques.

Interobserver reliability was assessed using measurements from six observers: the two primary observers and four additional colleagues. All six observers assessed the first 15 sequentially included patients for this reliability analysis. A two-way random-effects, average-measures intraclass correlation coefficient, ICC(2,6), was used. Intraobserver reliability was assessed using a two-way mixed-effects, single-measure intraclass correlation coefficient, ICC(3,1). ICCs were reported with 95% CIs. ICC values were interpreted according to Koo and Li: <0.50 poor, 0.50–0.75 moderate, 0.75–0.90 good and >0.90 excellent reliability [9].

### Statistical analysis

Continuous variables were summarised using means, standard deviations (SDs) and 95% CIs where appropriate. The paired difference was defined as short-leg minus standing long-leg β angle; a negative difference therefore indicated a more varus measurement on the short-leg radiograph. Agreement was assessed using the mean paired difference and Bland–Altman 95% limits of agreement. Absolute differences were categorised as ≤0.5°, >0.5–1°, >1–2° and >2° and were summarised descriptively.

For the prespecified hypothesis analysis, knees demonstrating varus on standing long-leg radiographs (*β* angle <90°) were compared using a one-sided paired *t*-test. A paired analysis was used because both measurements came from the same knee; a one-sided test was used because the priori hypothesis was directional. Knees demonstrating valgus on standing long-leg radiographs (*β* angle >90°) were evaluated in a subgroup analysis. Knees with a *β* angle of exactly 90° were classified as neutral and excluded from both subgroups. Statistical significance was defined as *p* < 0.05. Statistical analyses and figures were produced using R (R Core Team, 2024).

## Results

### Reliability analysis

Interobserver reliability was excellent. Using ICC(2,6), the ICC was 0.9994 (95% CI: 0.9988–0.9997) for short-leg radiographs and 0.9998 (95% CI: 0.9996–0.9999) for standing long-leg radiographs (both *p* < 0.0001). Intraobserver reliability was also excellent. Using ICC(3,1), the ICC was 0.9972 (95% CI: 0.9933–0.9999) for short-leg radiographs and 0.9980 (95% CI: 0.9951–0.9993) for standing long-leg radiographs (both *p* < 0.0001).

### Comparison of radiographic techniques

The mean *β* angle was 86.91° on short-leg radiographs and 88.04° on standing long-leg radiographs (Figure S1). The mean paired difference was −1.13° (95% CI: −1.40° to −0.85°), indicating that short-leg radiographs demonstrated a systematic shift towards a more varus measurement.

The absolute difference was categorised as ≤0.5°, >0.5–1°, >1–2° or >2° (Figure S2). Overall, 71% of measurements differed by ≤2°, whereas 29% differed by >2°.

Bland–Altman analysis demonstrated a mean difference of −1.13° (SD 1.51°) and 95% limits of agreement of −4.08° to 1.82° (Figure S3). No clear relationship between measurement magnitude and difference magnitude, including no obvious funnel-shaped distribution, was evident on visual inspection.

### Varus subgroup analysis

Forty-seven knees demonstrated varus alignment on standing long-leg radiographs. The paired measurements are presented in Figure S4. The mean paired difference was −0.79° (SD 1.33°; 95% CI: −1.18° to −0.40°). A one-sided paired t-test indicated that short-leg *β*-angle measurements were lower than the corresponding standing long-leg measurements (*t*(46) = −4.10; *p* < 0.001), supporting the prespecified directional hypothesis. The Bland–Altman 95% limits of agreement were −3.41° to 1.18° (Figure S5).

### Valgus subgroup analysis

Fourteen knees demonstrated valgus alignment on standing long-leg radiographs. One knee with a standing long-leg *β* angle of exactly 90.00° was classified as neutral and excluded. In this exploratory subgroup, the mean paired difference was −2.31° (SD 1.57°; 95% CI: −3.22° to −1.40°), indicating a larger mean shift towards varus on short-leg radiographs than in the varus subgroup (paired *t*-test: *t*(13) = −5.51; *p* < 0.001). Bland–Altman analysis yielded 95% limits of agreement of −5.39° to 0.76° (Figure S6), indicating substantial individual-patient variability.

## Discussion

The principal finding was that short-leg radiographs produced a systematic shift towards a more varus *β*-angle measurement compared with standing long-leg radiographs after TKA. The overall mean difference was −1.13°, and 29% of measurements differed by more than 2°. Most importantly, the Bland–Altman 95% limits of agreement extended from −4.08° to 1.82°. Thus, although the average difference was modest, substantially larger discrepancies occurred in individual patients, and the two techniques should not be considered interchangeable.

Several methods are available for assessing coronal alignment after TKA, including clinical examination, computed tomography, short- and long-leg radiographs, intraoperative fluoroscopy and computer-assisted navigation. Their accuracy and reproducibility vary, and no single method is a universal gold standard [5–8, 10]. Standing long-leg radiographs allow visualisation of the entire lower-limb mechanical axis and were therefore used as the reference technique in this study.

The definition and clinical significance of malalignment have become more complex with the development of different alignment philosophies. Deviation of more than 3° from neutral has traditionally been considered malalignment in mechanically aligned TKA [1], but this definition cannot necessarily be applied to kinematic, restricted kinematic or functional alignment techniques. Radiographic differences should therefore be interpreted in the context of the alignment philosophy used.

Patient satisfaction following primary TKA remains imperfect, and unmet expectations, residual pain and functional limitations contribute to dissatisfaction [11]. Coronal alignment may also influence outcomes: postoperative alignment has been associated with patient satisfaction [12], although the relationship between alignment, function and implant survivorship remains debated [10, 13–18].

Previous studies similarly reported discrepancies between short- and long-leg radiographs. Petterwood et al. observed differences of up to 5° in 9% of patients [2]. Park et al. found discordant varus, neutral or valgus classification in approximately one-third of postoperative TKAs [3]. Kazemi et al. reported substantially different proportions of neutral alignment on short-knee and full-length radiographs [4]. The present findings extend this evidence by quantifying both systematic bias and individual-level agreement.

The varus subgroup supported the prespecified hypothesis: short-leg radiographs demonstrated a mean shift of 0.79° towards varus. Although statistically significant, the mean magnitude was small. The valgus subgroup showed a larger mean shift of 2.31° towards varus, but the subgroup comprised only 14 knees and the limits of agreement were wide. This finding should therefore be interpreted cautiously and requires confirmation in a larger valgus cohort.

The 29% of measurements differing by more than 2° is clinically noteworthy, but 2° has not been established as a validated threshold for clinically important disagreement. The excellent interobserver and intraobserver reliability suggests that the discrepancy is unlikely to be explained solely by observer variability and instead relates, at least in part, to differences between the radiographic techniques.

Several limitations should be acknowledged. This retrospective study had a relatively small sample, particularly in the valgus subgroup. Although radiographs were obtained in one department using standardised protocols, positioning could not be fully standardised in the presence of fixed flexion contracture or severe deformity; limb rotation and flexion may therefore have affected the *β* angle. The cohort included TKAs performed according to different alignment philosophies, limiting application of a single definition of optimal alignment. Finally, standing long-leg radiographs were used as the reference technique but should not be regarded as a definitive gold standard for true postoperative alignment.

These findings support continued use of standing long-leg radiographs when accurate postoperative assessment of the mechanical axis is required. A fixed 1.13° correction should not be applied to individual short-leg measurements because the wide limits of agreement show that patient-level differences vary substantially. Future studies should evaluate the effects of limb rotation and flexion deformity and should investigate the larger discrepancy observed in valgus-aligned TKAs.

## Conclusion

Short-leg and standing long-leg radiographs demonstrate systematic differences in the assessment of coronal alignment following TKA. Short-leg radiographs showed a mean shift of 1.13° towards varus, while 29% of measurements differed by more than 2°. The comparatively wide 95% limits of agreement indicate that larger discrepancies may occur in individual patients. The mean shift was 0.79° in varus knees and 2.31° in the smaller valgus subgroup. Short-leg radiographs should not be considered interchangeable with standing long-leg radiographs; standing long-leg radiographs remain preferable when accurate assessment of the lower-limb mechanical axis is required.

## Figures and Tables

**Figure S1:**
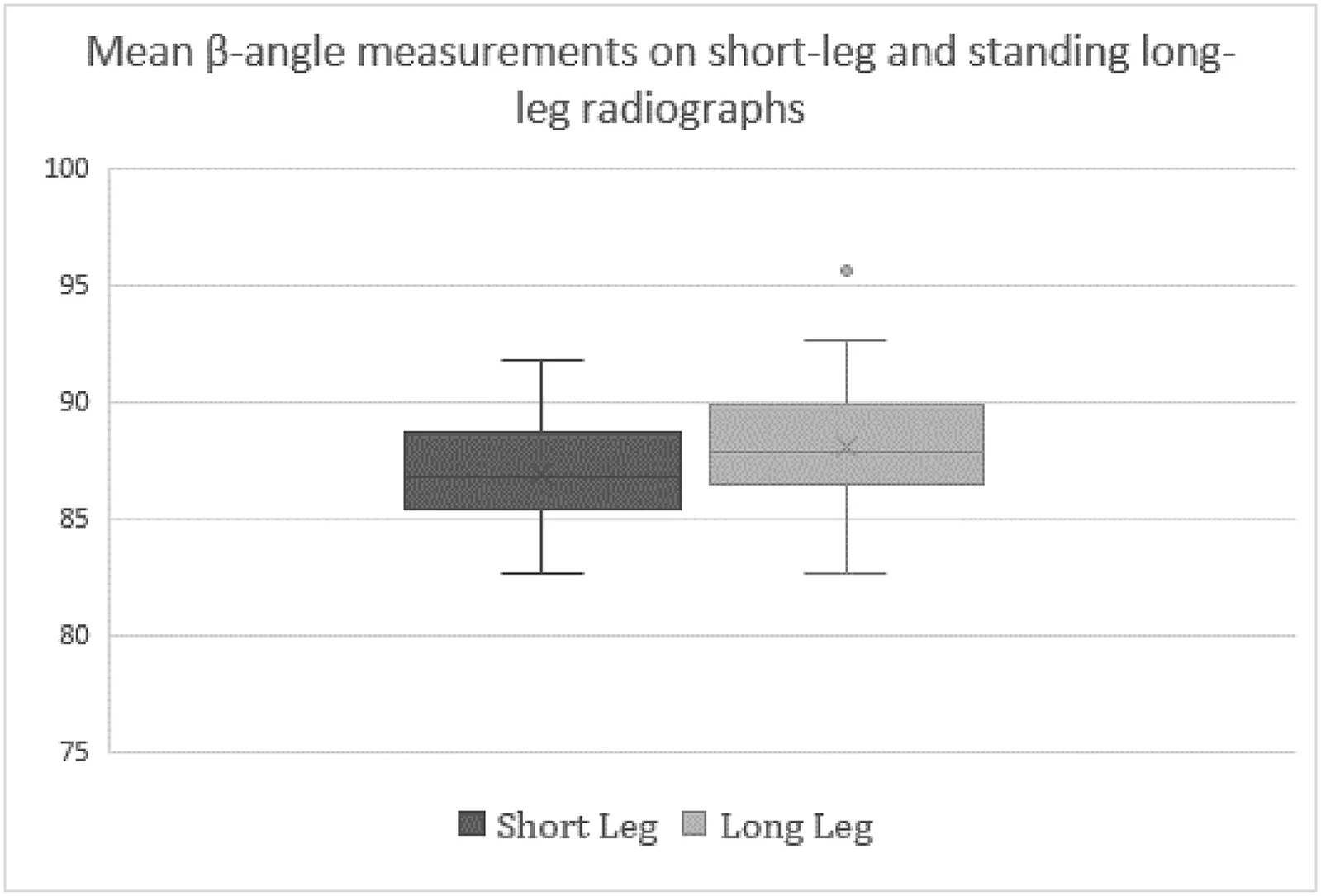
β-angle measurements on short-leg and standing long-leg radiographs.

**Figure S2:**
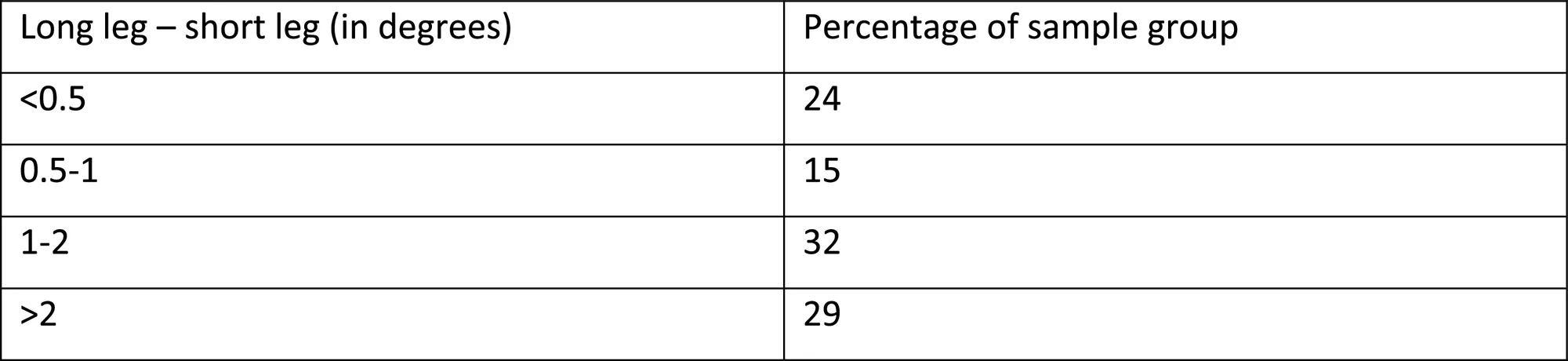
Distribution of absolute differences between paired β-angle measurements.

**Figure S3:**
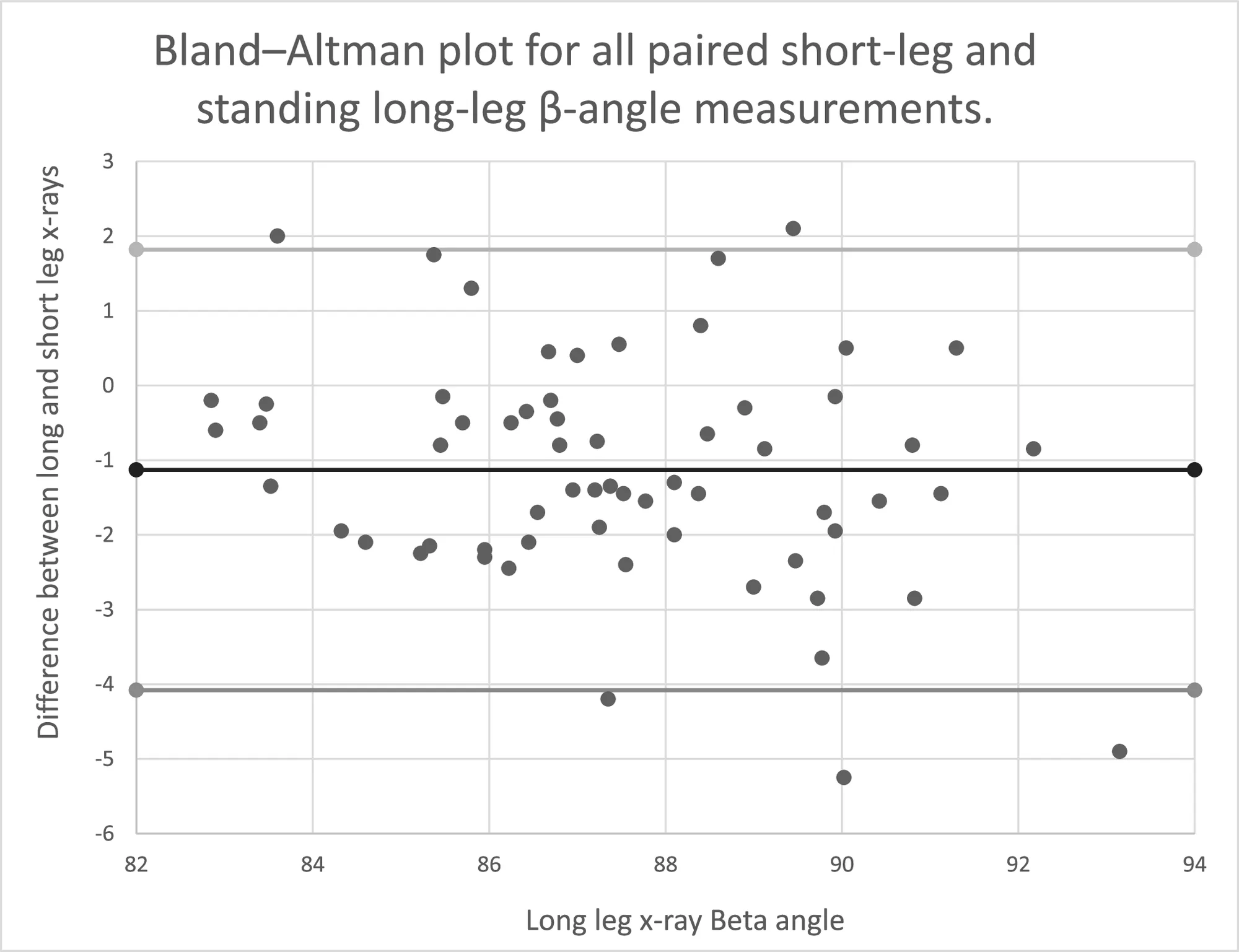
Bland–Altman plot of β-angle agreement in the full cohort.

**Figure S4:**
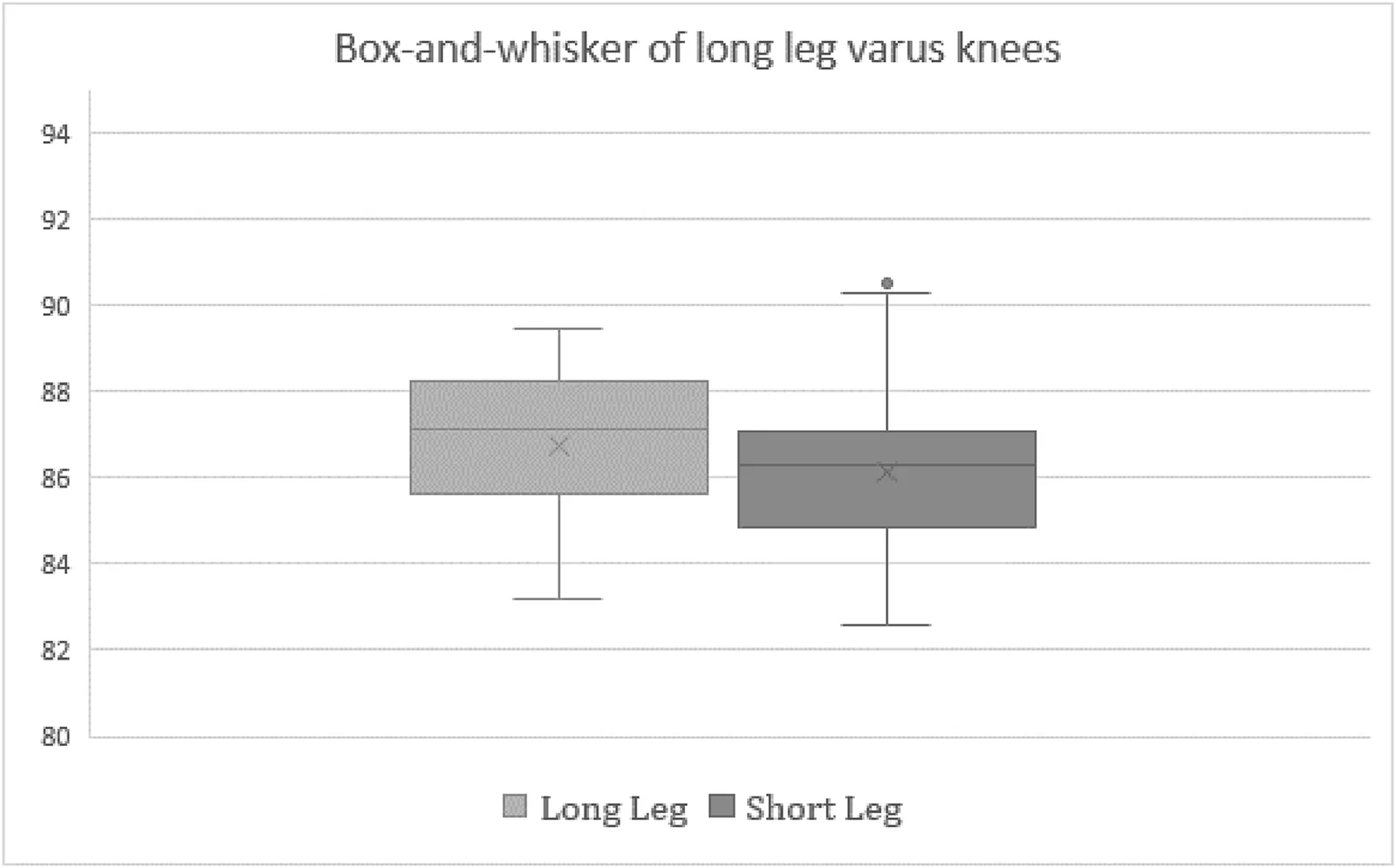
β-angle measurements on short-leg and standing long-leg radiographs in the varus subgroup.

**Figure S5:**
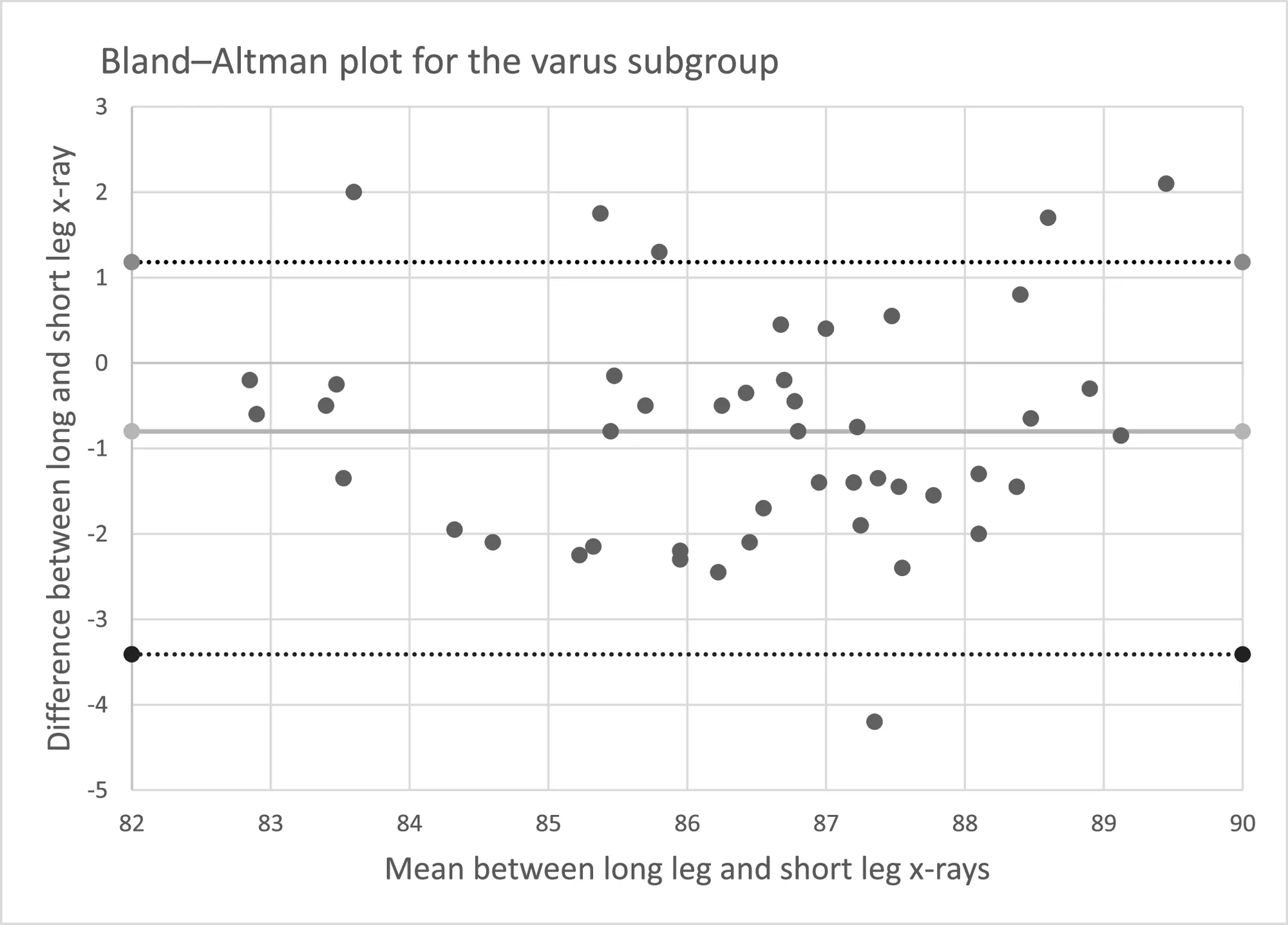
Bland–Altman plot of β-angle agreement in the varus subgroup.

**Figure S6:**
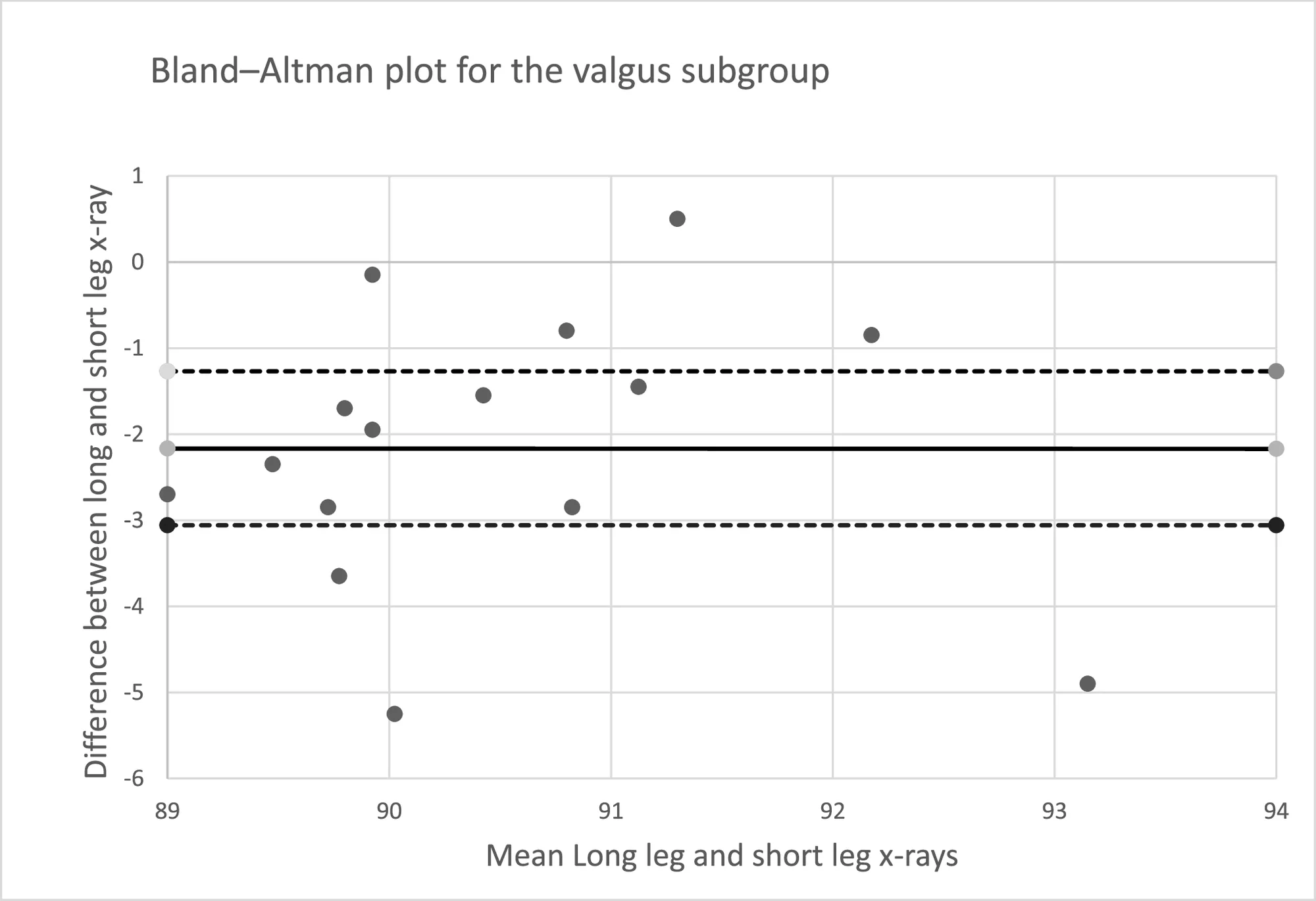
Bland–Altman plot of β-angle agreement in the valgus subgroup.

## Data Availability

The data supporting the findings of this study are available from the corresponding author on reasonable request, subject to applicable ethical and institutional requirements.

## Declaration of AI use

ChatGPT (OpenAI; GPT-5.6 Sol) was used during manuscript preparation to assist with language editing, grammatical revision, organisation, clarity and presentation of the reported statistical results. Codex (OpenAI; accessed on 8 September 2026) subsequently assisted with editing and formatting this article within the dissertation, incorporating the revised manuscript and aligning the surrounding thesis summaries with its findings. The Codex revision did not generate or alter study data or radiographic images and did not perform new statistical analyses. The authors remain responsible for reviewing and verifying AI-assisted content and for the accuracy, integrity and final interpretation of the manuscript.

## Supplementary material

The supplementary material of this article is available at https://www.sicot-j.org/10.1051/sicotj/2026079/olm.
